# *PIM-1* Is Overexpressed at a High Frequency in Circulating Tumor Cells from Metastatic Castration-Resistant Prostate Cancer Patients

**DOI:** 10.3390/cancers12051188

**Published:** 2020-05-08

**Authors:** Athina Markou, Eleni Tzanikou, Areti Strati, Martha Zavridou, Sophia Mastoraki, Evangelos Bournakis, Evi Lianidou

**Affiliations:** 1 Analysis of Circulating Tumor Cells, Lab of Analytical Chemistry, Department of Chemistry, University of Athens, 15771 Athens, Greece; atmarkou@chem.uoa.gr (A.M.); tzanikou.elena@windowslive.com (E.T.); artyzodim@gmail.com (A.S.); marthazavridou@hotmail.com (M.Z.); sophia.mastoraki88@gmail.com (S.M.); 2 Oncology Unit, 2nd Department of Surgery, Aretaieio Hospital, Medical School, National and Kapodistrian University of Athens, V. Sophias 76, 11528 Athens, Greece; vagimith@yahoo.com

**Keywords:** liquid biopsy, CTCs, mCRPC, *PIM-1*, *AR-V7*, RT-qPCR

## Abstract

*PIM-1* is an oncogene involved in cell cycle progression, cell growth, cell survival and therapy resistance, activated in many types of cancer, and is now considered as a very promising target for cancer therapy. We report for the first time that *PIM-1* is overexpressed in circulating tumor cells (CTCs) from metastatic castration-resistant prostate cancer patients (mCRPC). We first developed and validated a highly sensitive RT-qPCR assay for quantification of *PIM-1* transcripts. We further applied this assay to study *PIM-1* expression in EpCAM^(+)^ CTC fraction isolated from 64 peripheral blood samples of 50 mCRPC patients. CTC enumeration in all samples was performed using the FDA-cleared CellSearch^®^ system. *PIM-1* overexpression was detected in 24/64 (37.5%) cases, while in 20/24 (83.3%) cases that were positive for *PIM-1* expression, at least one CTC/7.5 mL PB was detected in the CellSearch^®^. Our data indicate that *PIM-1* overexpression is observed at high frequency in CTCs from mCRPC patients and this finding, in combination with androgen receptor splice variant 7 (AR-V7) expression in CTCs, suggest its potential role as a very promising target for cancer therapy. We strongly believe that *PIM-1* overexpression in EpCAM^(+)^ CTC fraction merits to be further evaluated and validated as a non-invasive circulating tumor biomarker in a large and well-defined patient cohort with mCRPC.

## 1. Introduction

Prostate cancer (PCa) is the second most common cancer in men worldwide, with an estimated global incidence of 1.3 million cases in 2018 [1]. Therapeutic options exist for patients with clinically localized disease, and the 10-year survival rate is over 90% [2]. However, a significant minority of patients present de novo metastatic disease after initiation of primary treatment with androgen deprivation therapy (ADT). In most cases, the progression is inevitable leading to increasing values of serum prostate-specific antigen (PSA) despite the castrated levels of serum testosterone (<50 ng/dL), termed the disease state as castration-resistant prostate cancer (CRPC) [3,4]. In recent years, the therapeutic management of advanced disease has been rapidly improved, including mainly taxanes as chemotherapy regimens such as docetaxel and cabazitaxel and androgen receptor (AR) inhibitors such as abiraterone acetate and enzalutamide, significantly increasing the life expectancy of metastatic castration-resistant prostate cancer (mCRPC) patients [5]. The diagnosis and monitoring of PCa are currently based on the combination of PSA testing, abnormal digital rectal examination and histopathologic evaluation of prostate biopsy [6]. Although PSA became widely adopted for cancer screening by the early 1990s, its main drawback is the lack of specificity and its limited value for predicting responses to therapy [7]. Since classical biopsy is highly invasive, it cannot be used to monitor tumor genomic changes in real time.

Liquid biopsy, based on serial blood testing, covers this gap by enabling the prospective and sequential evaluation of the disease dynamics, and this is feasible for the detection of minimal residual disease and early prediction of relapse [8,9]. Liquid biopsy is based on the analysis of circulating tumor cells (CTCs), circulating tumor DNA (ctDNA), circulating miRNAs and tumor-derived extracellular vesicles (EVs) that are shed from primary tumors or metastatic sites into peripheral blood [9,10,11]. The test for CTC enumeration in metastatic prostate cancer is FDA-cleared for prognosis since 2008 [12,13]. In addition to CTC enumeration, the molecular characterization of CTC in mCRPC has important therapeutic implications; androgen receptor splice variant 7 (AR-V7) expression in CTCs from patients with mCRPC predicts a lack of response to anti-androgen therapy with enzalutamide or abiraterone [14], while AR-V7 expression status does not affect responsiveness to taxanes [15]. We have recently developed and validated a multiplex RT-qPCR assay for AR splice variants and have shown that the AR-V7 splice variant is highly overexpressed in CTCs of patients with mCRPC [16].

However, new surrogate biomarkers that could be easily measurable and could predict the treatment outcomes for prostate cancer management are still highly needed. Basic research has shown that the proviral integration site for the Moloney murine leukemia virus-1 (*PIM-1*) is an oncogene that encodes a serine/threonine kinase, involved in cell cycle progression, cell growth, cell survival and therapy resistance [17,18]. *PIM-1* is activated in many types of cancer including prostate, providing a common target for therapy [19,20,21]. Recent data have shown that PIM activation is induced by tumor microenvironment changes, such as hypoxia, and causes resistance to angiogenesis inhibitors [21]. *PIM-1* is a component of the small 40S ribosomal subunit and could regulate the expression of ribosomal small subunit protein-7, RPS7, demonstrating that ribosome-targeting drugs may be effective against diverse CRPC subtypes including AR-null disease [22,23]. Moreover, PIM-1 is thought to promote the carcinogenesis by cooperating with myc as transgenic mouse study has demonstrated that PIM1 enhanced c-Myc-induced tumorigenesis in PCa [24]. *PIM-1* has been shown to be overexpressed in approximately 50% of human prostate cancer specimens using tissue microarrays [25]. Moreover, *PIM-1* overexpression was observed in high-grade prostate intraepithelial neoplasia and in prostate cancer compared to normal prostatic tissue and benign prostate hyperplasia [26,27]. Increased levels of *PIM-1* have been shown to be the direct result of oncogenic fusion proteins and active signal transduction pathways, while its elevated levels can lead to genomic instability and promote the neoplastic process [28]. *PIM-1* kinase can also phosphorylate *AR*, regulating its degradation and function, indicating its involvement in mCRPC. Furthermore, *PIM-1* expression has been shown to be increased in prostate tissue demonstrating partial response to docetaxel, suggesting the predictive role of *PIM-1* to this type of treatment [28]. Initial efforts to inhibit *PIM* with monotherapies have been hampered by compensatory upregulation of other pathways and drug toxicity, and as such, it has been suggested that co-targeting *PIM* with other treatment approaches may permit lower doses and be a more viable option in the clinic [29].

In this study, we first developed and validated a highly sensitive RT-qPCR assay for quantification of *PIM-1* transcripts and reported for the first time that *PIM-1* is overexpressed in EpCAM^(+)^ CTC fraction isolated from mCRPC patients. We further evaluated whether *PIM-1* overexpression in EpCAM^(+)^ CTC fraction is correlated with ARV7 expression in the same samples. Our data indicate that *PIM-1* overexpression in CTCs should be prospectively evaluated as a potential biomarker for prostate cancer management in a large and well-defined patient cohort.

## 2. Results

**Figure 1:**
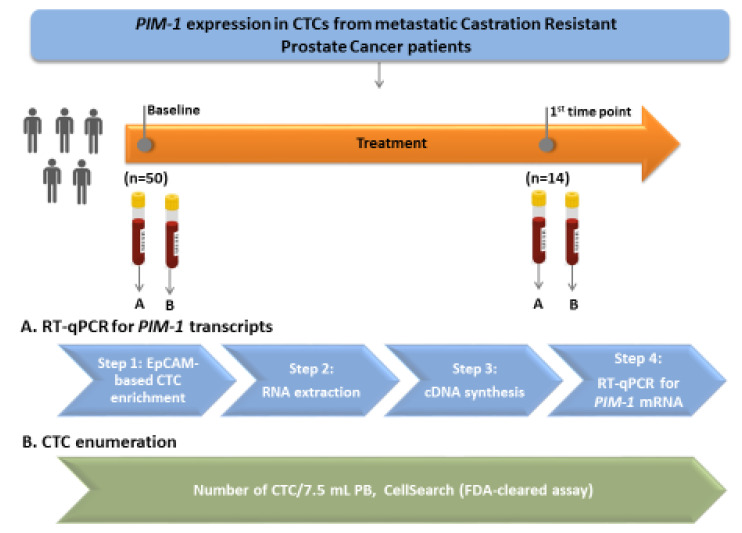
Outline of the experimental procedure.

The outline of the study is shown in Figure 1.

### 2.1. TCGA Analysis

In The Cancer Genome Atlas (TCGA), the PanCancer Atlas for the prostate cohort contains data from 492 prostate adenocarcinoma patients (PRAD). Bioinformatic analyses of the TCGA datasets demonstrated that *PIM-1* is elevated in 28/492 (6%) cases. To verify *PIM-1* mRNA expression, the GEPIA (http://gepia.cancer-pku.cn/index.html) web server was used to plot a gene expression level between prostate adenocarcinoma and normal tissues in the TCGA database (Figure S1). The patient data were grouped according to the transcripts per million (TPM) value. Log2 (TPM + 1) was used for log-scale, and four-way analysis of variance (ANOVA) was applied.

### 2.2. PIM-1 Overexpression in EpCAM^(+)^ CTC Fraction

**Figure 2:**
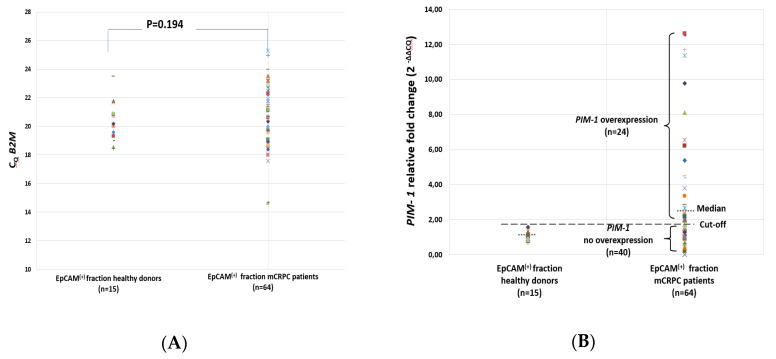
(**A**) Cq values for *B2M* and (**B**) relative fold change for *PIM-1* in the EpCAM^(+)^ fraction in healthy donors (HD) (*n* = 15) and metastatic castration-resistant prostate cancer (mCRPC) patients samples (*n* = 64).

A total of 64 peripheral blood samples from 50 mCRPC patients collected at two different time points were used to isolate EpCAM(+) fractions, isolate total RNA and synthesize cDNAs. All these cDNAs were first checked for their quality by RT-qPCR for B2M. All these cDNA samples were positive for B2M expression. B2M expression levels did not differ between EpCAM^(+)^ fractions in the mCRPC patients group and the healthy donors (HD) group, as expected (Figure 2A). In these cDNAs, we performed RT-qPCR to quantify *PIM-1* expression in the EpCAM^(+)^ fractions.

A novel method based on RT-qPCR for *PIM-1* assay was developed and the experimental conditions were first optimized in detail. Under optimized conditions, the specificity of the assay was tested using peripheral blood samples from 15 healthy donors (HD) that were analyzed exactly as patient samples [16]. Median fold change of *PIM-1* expression in the HD group was used to define the cut-off (1.03, range: 0.7–1.58). Based on the defined cut-off, 40/64 (62.5%) patient samples were found negative for *PIM-1* overexpression (median fold change: 0.98, range: 0.04–1.51, *p* = 0.034) and 24/64 (37.5%) samples were found positive for *PIM-1* overexpression (median fold change: 5.13, range: 1.53–12.64, *p* < 0.001) (Figure 2B). *PIM-1* overexpression was detected in 21/50 (42%) samples at baseline (before) and in 3/14 (21.4%) samples at the first time point of treatment (after).

### 2.3. PIM-1 Overexpression in the EpCAM^(+)^ CTC Fraction before and after Treatment

**Figure 3:**
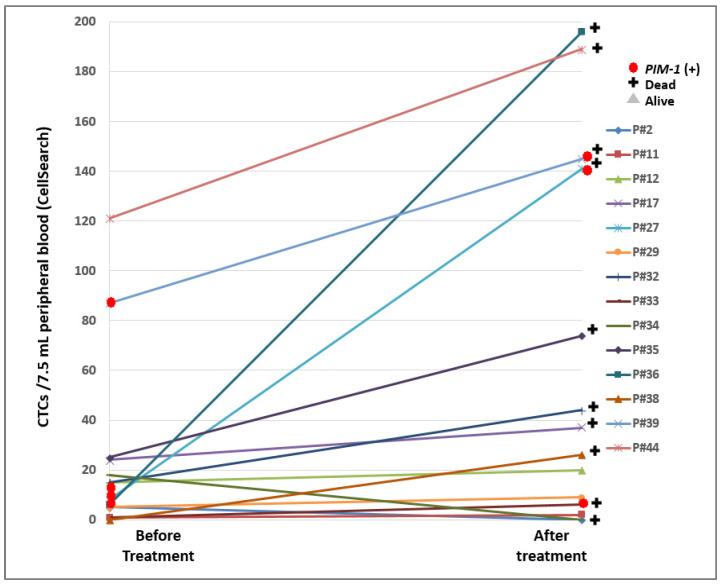
CTC enumeration (CTCs/7.5 mL PB, CellSearch^®^) in 14 pairs of mCRPC patient samples before and after treatment and *PIM-1* overexpression in EpCAM^(+)^ CTC fraction.

For a subgroup of these mCRPC patients (*n* = 14), PB samples were available both at baseline and at the first time point of treatment. In this group, *PIM-1* overexpression was observed in total of 7/28 (25%) EpCAM^(+)^ CTC fraction samples; in 25/28 (89.3%) of these cases CTCs were detected by the CellSearch^®^, and more than 5CTCs/7.5 mL were identified in 22/25 (88%) of CTC-positive samples (Table 1). There was only one case (P#38) where the EpCAM^(+)^ CTC fraction was found to be positive for *PIM-1* overexpression, whereas CellSearch^®^ didn’t identify CTCs. There were two cases (P#2, P#34) where both CellSearch^®^ and *PIM-1* expression analyses were negative (Table 1). It is important to note that 5/14 (35.7%) patient samples (P#2, P#27, P#33, P#38, P#39) were positive for *PIM-1* overexpression, in at least one time point of treatment, and that 4/5 (80%) of these patients where *PIM-1* was overexpressed in CTCs have died (Table 1). There was only one case (P#2) where the patient was identified as positive for *PIM-1* overexpression at baseline and was still alive at the time of our results evaluation (Table 1, Figure 3).

### 2.4. PIM-1 Overexpression in Relation to CTC Enumeration in the CellSearch^®^ System

**Figure 4:**
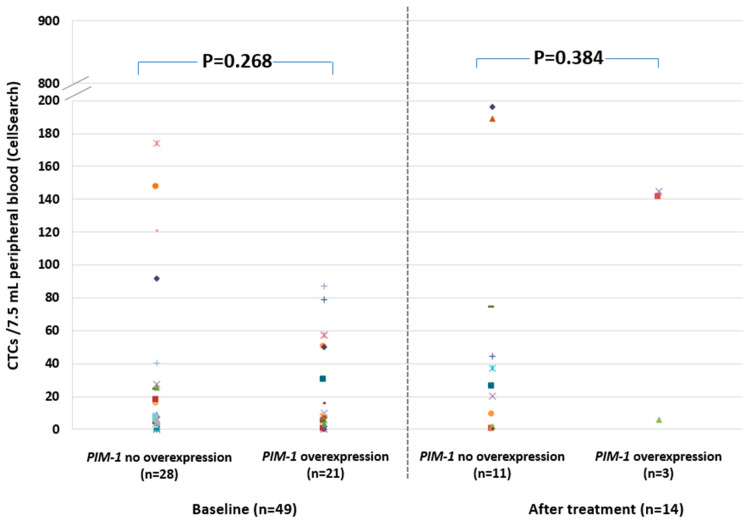
Relative fold change (2^−ΔΔCq^) of *PIM-1* transcripts in EpCAM^(+)^ CTC fraction before and after treatment, in relation to CTC enumeration (CTCs/7.5 mL PB, CellSearch^®^), in identical blood draws.

CTC enumeration was performed in parallel, in identical peripheral blood draws in 63 patient samples using the FDA-cleared CellSearch^®^ system (Menarini, Silicon Biosystems), 49 at baseline, and 14 after the first time point of treatment. CTCs were detected by the CellSearch^®^ in 53/63 (84.1%) cases, while in 44/63 (69.8%) cases at least 5CTCs/7.5mL PB were enumerated. *PIM-1* was overexpressed in 21/49 (42.8%) of these samples before and in 3/14 (21.4%) samples after the first time point of treatment (Figure 4). In 20/53 (37.7%) cases where the CellSearch^®^ analysis detected at least one CTC/7.5mL PB, *PIM-1* was found to be overexpressed. However, there were four cases where EpCAM^(+)^ CTC fractions were found positive for *PIM-1* overexpression, while in the CellSearch^®^ no CTCs were detected. According to our results *PIM-1* overexpression in CTC was not associated with CTC counts both before and after treatment (Figure 4). *PIM-1* overexpression was detected in 24/63 (38.1%) cases; 21/49 (42.8%) were positive for *PIM-1* overexpression before treatment and 3/14 (21.4%) were positive for *PIM-1* overexpression after the first time point of treatment. It is important to mention that in the majority 20/24 (83.3%) of these samples that were positive for *PIM-1* overexpression, at least one CTC/7.5 mL PB was detected in the CellSearch^®^ (Figure 4).

### 2.5. PIM-1 Overexpression in Relation to AR-V7 Expression

We further evaluated for the first time whether *PIM-1* overexpression in EpCAM^(+)^ CTC fraction is correlated with *AR-V7* expression in the same samples. For 44/50 (88%) of these patients, the status of *AR-V7* expression in EpCAM^(+)^ CTCs before treatment was known to us through our previous study [16]. Our comparison indicated that 5/44 (11.4%) samples were positive for both *PIM-1* overexpression and *AR-V7* expression; 4/5 (80%) of these patients died (Table S1). There were 13/44 (29.5%) samples positive for *PIM-1* overexpression and negative for AR-V7 expression, and 10/44 (22.7%) samples positive for *AR-V7* expression and negative for *PIM-1* overexpression (Table S1). Thus, in total, in 28/44 (63.6%) patient samples either *PIM-1* was overexpressed or/and *AR-V7* was positive in EpCAM^(+)^ CTC fraction, and a high percentage (20/28, 71.4%) of these patients died. On the contrary, 10/16 (62.5%) patients, where in the EpCAM^(+)^ CTC fraction *PIM-1* was not overexpressed and *AR-V7* was also negative, were still alive (Table 2). According to these findings, although there was no association between *PIM-1* overexpression in EpCAM^(+)^ fraction and the outcome of the patients (*p* = 0.296), there was a statistically significant association between *PIM-1* overexpression and/or expression of *AR-V7* in EpCAM^(+)^ fraction before treatment and the outcome of the same patients (chi-square *p* = 0.030).

## 3. Discussion

The application of liquid biopsy in metastatic prostate cancer has been the most rapidly evolving paradigm of translational research in recent years. In metastatic prostate cancer, CTC-enumeration is an established and FDA-cleared prognostic test that allows the estimation of overall metastatic burden in cancer patients. Beyond enumeration, the molecular characterization of CTCs hold great promise to improve our knowledge of the metastatic process and to identify new treatment predictive markers. At present, two commercial AR-V7 detection systems are available for clinical use in order to guide which patients will benefit from enzalutamide or abiraterone treatment. CTC molecular analysis based on *AR-V7* transcript in mCRPC patients was first described by RT-qPCR performed on EpCAM immuno-magnetically captured cells using the AdnaTest platform (Qiagen, Hilden, Germany). The Oncotype DX *AR-V7* Nucleus Detect Test, which is the second platform, was developed by Epic Sciences (Epic Science, San Diego, California), and is based on immunofluorescent CTC staining [30,31]. Detection of *AR-V7* at the protein level by this test led to the first approval of CTCs as a predictive biomarker to guide the choice of therapy [9,29]. It is also important to note that a more recent comparison study between these two assays, performed under the PROPHECY trial, has demonstrated a very good agreement (82%) [32].

It is now clear in many cancer types, that as CTCs constitute a dynamic heterogenic population of cancer cells from several primary or metastatic lesions, changes at the gene expression [16,33,34], DNA methylation [35,36,37,38], and DNA mutation levels [39,40,41,42] do occur during treatment. These molecular changes can be evaluated for their potential as novel biomarkers in prostate cancer. Recently, after developing highly sensitive multiplex RT-qPCR assays for the expression of 14 genes, we have shown that the combination of in vivo CTC isolation with downstream RNA analysis is highly promising as a high-throughput, specific, and ultrasensitive approach for multiplex liquid biopsy-based molecular diagnostics in prostate cancer [33].

In the present study, we evaluated for the first time *PIM-1* overexpression in the EpCAM^(+)^ fraction of mCRPC patients using a highly sensitive and specific RT-qPCR assay. We have chosen to study *PIM-1*, since very recent data have shown that *PIM-1* kinase plays a critical role in tumorigenesis, and overexpression of *PIM-1* protein has been suggested as a potential biomarker for many malignancies including prostate cancer [43]. In preclinical studies, *PIM-1* overexpression may lead to cancer development in the following major ways; by inhibiting apoptosis, by promoting cell proliferation and also through promoting genomic instability [44].

Our results indicate that *PIM-1* is overexpressed at a high frequency in EpCAM^(+)^ CTC fraction in mCRPC (37.5%), when compared to the TCGA data in prostate adenocarcinoma primary tumors (6%). In the present study, we report for the first time that *PIM-1* is overexpressed in CTCs. This positivity rate (37.5%) is not only detected for the first time in CTCs, but in comparison to other genes tested for their expression in EpCAM^(+)^, CTCs’ expression in similar samples [16] is really very high. When we compared *PIM-1* overexpression in CTCs with the corresponding CTC counts as estimated by CellSearch^®^, we found that it was not associated with CTC counts both before and after treatment. We found that in a substantial number of cases where CellSearch^®^ detected CTCs, these CTCs were positive for *PIM-1* overexpression. However, there were four cases where CTCs were not detected by the CellSearch^®^, but the corresponding EpCAM^(+)^ fractions were positive for *PIM-1* overexpression by RT-qPCR. These results could be possibly explained by the fact that some EpCAM-positive cells can be negative for CKs (CK-8, CK-18, CK-19) due to epithelial–mesenchymal transition (EMT) process, so they are reported as negative for CTC by the CellSearch^®^. These findings are in accordance with our previous studies where we detected a lot of molecular alterations in EpCAM^(+)^ CTC fraction, in samples that were “officially” negative for CTCs when using the CellSearch^®^ system [33,41,42].

In recent years, PIM kinase has become one of the important therapeutic targets for the development of novel cancer therapeutics and many inhibitors are under different phases of clinical trials [43,45,46]. Several different derivatives have been synthesized and evaluated for their PIM inhibitory activity, including pyrrole [47,48], pyrimidine [49,50], thiazolidine [51], indole [52], triazole [53], oxadiazole [54], and quinolone [55]. All these derivatives have a specific ring or functional groups which are associated with the PIM kinase inhibitory activity. It is highly important to note that according to our results, in most cases *PIM-1* overexpression in EpCAM^(+)^ CTCs at least in one time point during treatment, was associated with the death of patients. This finding indicates that therapy targeted towards *PIM-1* would inhibit the activation of this molecule, and could possibly lead to a better clinical outcome for these patients [29].

It is well known that androgen receptor plays a crucial role in the regulation of the normal prostate as well as in the promotion and progression of prostate cancer. However, many studies have investigated whether the regulation of AR transcriptional activity by post-translational modifications, such as phosphorylation, is affected by multiple kinases. PIM1 is a kinase that is overexpressed in prostate cancer, while the two isoforms, PIM-1S and PIM-1L, are the major mediators of AR serine 213 (Ser-213) and threonine 850 (Thr-850) phosphorylation. Based on our previous published study regarding the evaluation of *AR-V7* molecular profile in CTCs, we proceed further to co-evaluate for the first time the pattern status of *PIM-1* overexpression and/or *AR-V7* in the same samples [16]. We noticed that the majority (74.1%) of patients where *PIM-1* was overexpressed or/and *AR-V7* was positive in the EpCAM^(+)^ CTC fractions have died. On the contrary, the majority (62.5%) of patients where *PIM-1* was not overexpressed and *AR-V7* was negative in the EpCAM^(+)^ CTCs fractions were still alive at the time of analysis of our data. Our findings are in accordance with the very recent study of Luszczak et al., who demonstrated that AR levels do not appear to affect *PIM-1*, suggesting that the combination of PIM inhibitors and androgen deprivation therapy are needed in order to assess whether the inhibition of *PIM-1* could overcome resistance to androgen deprivation therapy [29].

## 4. Materials and Methods

### 4.1. Clinical Samples

We analyzed 64 peripheral blood samples (20 mL in EDTA) from 50 patients with mCRPC; 50 samples at baseline, before treatment, and for 14 patients a sample at the first time point of treatment was available, and 15 peripheral blood samples from healthy male donors. In 63/64 of these cases, using the same blood draw, PB (7.5 mL) was isolated in CellSave tubes for CTC enumeration in the FDA-cleared CellSearch^®^ system (Menarini, Silicon Biosystems, Italy) [56]. The first 5 mL were not used, to avoid contamination from skin epithelial cells. For 14 patients, peripheral blood samples were also available at the first time point of treatment with abiraterone or enzalutamide. All patients gave a written informed consent to participate in the study, which was approved by the Ethics and Scientific Committee of Aretaieio University Hospital.

### 4.2. CTC Enumeration in the CellSearch^®^

For CellSearch^®^, 7.5 mL of venous blood was collected into CellSave tubes (Menarini, Silicon Biosystems) and processed using the CellSearch^®^ Circulating Tumor Cell Kit (Menarini, Silicon Biosystems) according to the manufacturer’s instructions.

### 4.3. CTC Immunomagnetic Enrichment and RNA-Based Analysis

EpCAM^(+)^ CTCs were enriched from 20mL peripheral blood in EDTA, using immune-magnetic capture beads coated with Ber-EP4 (Dynabeads^®^ Epithelial Enrich, Invitrogen) as previously described [56]. RNA and cDNA synthesis was performed as previously described [16,56].

### 4.4. RT-qPCR Assay for PIM-1 Expression

We first designed in-silico the primers and one hydrolysis probe (TaqMan) for *PIM-1* mRNA using Primer Premier 5.0 software (Premier Biosoft, San Francisco, CA, USA). Our primers and probe were carefully designed to completely avoid primer–dimer formation, false priming sites, formation of hairpin structures, and hybridization to genomic DNA, while amplifying specifically only *PIM-1* isoform according to our search in the BLAST Sequence Similarity Search tool (NCBI, NIH) (sequences available upon request). The hydrolysis probe included a 5′-fluorescein (FAM) as a fluorophore covalently attached to the 5’-end of the oligonucleotide probe and a Black Hole Quencher as a quencher at the 3’-end. *B2M* (Beta-2 microglobulin) was used as a reference gene. RT-qPCR was performed in the LightCycler^®^ 480 instrument (Roche, Germany). Detailed optimization experiments were carried out. The amplification reaction mixture for *PIM-1* contained 2 μL of the PCR synthesis buffer (5×), 1 μL MgCl_2_ (25 mM), 0.2 μL dNTPs (10 mM), 0.15 μL BSA (10 μg/μL), 0.1 μL Hot-Start DNA polymerase (Promega), 0.3 μL of forward and reverse primer (10 μΜ), 1 μL hydrolysis probe (3 μM) and H2O to a final volume of 10 μL, while the amplification reaction mixture for B2M contained 1 μL of PCR synthesis buffer (5×), 1.2 μL MgCl_2_ (25 mM), 0.15 μL dNTPs (10 mM), 0.3 μL BSA (10 μg/μL), 0.1 μL Hot Start DNA polymerase (Promega), 0.25 μL of forward and reverse primer (10 μΜ), 0.83 μL hydrolysis probe (3 μM) and H2O to a final volume of 10 μL.

### 4.5. RT-qPCR Assay for AR-V7 Expression

*AR-V7* expression in exactly the same cDNAs derived from EpCAM^(+)^ CTC fraction before therapy was evaluated in 44 of these 50 patients as previously described [16].

### 4.6. Quality Control

Each experimental procedure included one positive and one negative control. cDNA from PC3 cell line was used as a positive control. In order to ensure that amplification of gDNA was completely avoided, four genomic DNAs at high concentrations were used as templates. None of these DNA samples were amplified. B2M was used as a reference gene for RT-qPCR.

### 4.7. Statistical Analysis

RT-qPCR data for *PIM-1* expression were normalized in respect to *B2M* expression in the same cDNAs, using the 2^−ΔΔCt^ approach [57]. CTCs isolated through positive immune-magnetic enrichment are not 100% pure; since the presence of co-isolated PBMC in the EpCAM^(+)^ fraction could affect the specificity of the *PIM-1* assay, we evaluated this ‘background noise’ by analyzing peripheral blood samples from 15 healthy male individuals in exactly the same way as patients. We estimated a cut-off based on *PIM-1* normalized expression in respect to *B2M* expression in this control group (cut-off ΔΔCq = 1.51). Using this approach we defined a sample as positive *PIM-1* for overexpression (*PIM-1* positive) based on the fold change of *PIM-1* expression in the EpCAM^(+)^ fraction in respect to the corresponding EpCAM^(+)^ fraction in the group of these 15 healthy individuals.

## 5. Conclusions

We conclude that *PIM-1* overexpression is observed at high frequency in CTCs from mCRPC patients and this finding, in combination with *AR-V7* expression in CTCs, suggests its potential role as a very promising target for cancer therapy. Our data point towards the direction of prospective further evaluation of *PIM-1* mRNA overexpression in CTCs as a potential liquid biopsy-based biomarker in a large and well-defined cohort of mCRPC patients.

## Figures and Tables

**Table 1:** *PIM-1* overexpression in relation to circulating tumor cell (CTC) enumeration in the CellSearch^®^ system, Androgen Receptor splice variant 7 (*AR-V7*) expression and clinical outcome before and after treatment (*n* = 14).

Patient’s ID	CTCs/7.5 mL PB (CellSearch^®^ Analysis)		*PIM-1* in CTCs		*AR-V7* in CTCs		Therapy	Clinical Outcome	Death
	Before Therapy	After Therapy	Before Treatment	After Treatment	Before Treatment	After Treatment			
P#2	5	0	+	-	+	-	Enzalutamide	SD	No
P#11	1	2	-	-	-	-	Enzalutamide	CR	No
P#12	15	20	-	-	-	+	Docetaxel	PR	No
P#17	24	37	-	-	+	-	Docetaxel	PR	Yes
P#27	8	141	+	+	-	+	Docetaxel	PR	Yes
P#29	5	9	-	-	-	-	Abiraterone	CR	No
P#32	15	44	-	-	+	-	Docetaxel	PR	Yes
P#33	1	6	-	+	-	-	Abiraterone	PR	Yes
P#34	18	0	-	-	-	+	Abiraterone	PD	Yes
P#35	25	74	-	-	-	+	Docetaxel	PR	Yes
P#36	6	196	-	-	-	+	Docetaxel	PR	Yes
P#38	0	26	+	-	+	+	Docetaxel	PR	Yes
P#39	87	145	+	+	+	-	Docetaxel	PD	Yes
P#44	121	189	-	-	+	+	Abiraterone	PD	Yes

SD: Stable Disease, CR: Complete Response, PR: Partial Response, PD: Progression of Disease.

**Table 2:** Association between *PIM-1* overexpression and/or *AR-V7* expression in EpCAM^(+)^ CTC fraction and patient status in mCRPC patients (*n* = 44).

*PIM-1* Overexpression and/or *AR-V7* Expression	Patient Status		Total
	Alive	Dead	
NO	10 (62.5%)	6 (37.5%)	16
YES	8 (28.6%)	20 (71.4%)	28
Total	18 (40.9%)	26 (59.1%)	44
	Chi-square *p* = 0.030		

## Supplementary Materials

The following are available online at https://www.mdpi.com/2072-6694/12/5/1188/s1, Figure S1: *PIM-1* expression in Prostate Adenocarcinoma Tumors (*n* = 492) and normal prostate tissues (*n* = 52) according to the TCGA, Table S1: Association between *PIM-1* overexpression and *AR-V7* expression levels in EpCAM^(+)^ CTCs before treatment and clinical outcome of mCRPC patients (*n* = 44).

## Abbreviations

ADT	Androgen Deprivation Therapy
AR	Androgen Receptor
AR-V7	Androgen Receptor splice variant 7
CTCs	Circulating Tumor Cells
CtDNA	Circulating tumor DNA
EMT	Epithelial Mesenchymal Transition
EVs	Extracellular Vesicles
HD	Healthy Donors
CRPC	Castration-Resistant Prostate Cancer
PB	Peripheral Blood
PCa	Prostate Cancer
PRAD	Prostate Adenocarcinoma
TPM	Transcripts Per Million
