# Supplementary material for: PIM-1 Is Overexpressed at a High Frequency in Circulating Tumor Cells from Metastatic Castration-Resistant Prostate Cancer Patients
Source: Cancers (Basel). 2020 May 8;12(5):1188. doi: 10.3390/cancers12051188 (PMC7281625; doi:10.3390/cancers12051188)
Supplement: Supplementary file 1 [file cancers-12-01188-s001.pdf]

PIM-1 is Overexpressed at a High Frequency in Circulating Tumor Cells from Metastatic Castration-Resistant Prostate Cancer Patients

Athina Markou, Eleni Tzanikou, Areti Strati, Martha Zavridou, Sophia Mastoraki, Evangelos Bournakis and Evi Lianidou

Table S1. Association between *PIM-1* overexpression and *AR-V7* expression levels in EpCAM$^{(+)}$ CTCs before treatment and clinical outcome of mCRPC patients (n = 44).

	Patient #ID	*PIM-1*	*AR-V7*	Therapy	Clinical outcome	Death
1	P#1	-	+	Abiraterone	CR	YES
2	P#2	+	-	Enzalutamide	SD	NO
3	P#5	-	-	Enzalutamide	CR	NO
4	P#6	+	-	Enzalutamide	PR	YES
5	P#9	-	+	Enzalutamide	CR	YES
6	P#10	+	-	Docetaxel	PD	YES
7	P#11	-	-	Enzalutamide	CR	NO
8	P#12	-	-	Docetaxel	PR	NO
9	P#14	+	-	Docetaxel	PR	YES
10	P#15	-	+	Enzalutamide	CR	NO
11	P#16	-	+	Enzalutamide	PD	YES
12	P#17	-	+	Docetaxel	PR	YES
13	P#18	+	-	Enzalutamide	PD	YES
14	P#19	-	-	Enzalutamide	PR	NO
15	P#20	+	-	Enzalutamide	CR	NO
16	P#21	-	+	Docetaxel	PR	YES
17	P#22	+	-	Enzalutamide	PD	NO
18	P#23	-	-	Docetaxel	CR	YES
19	P#26	+	+	Enzalutamide	PD	YES
20	P#27	+	-	Docetaxel	PR	YES
21	P#28	+	-	Abiraterone	CR	NO
22	P#29	-	-	Abiraterone	CR	NO
23	P#30	-	+	Docetaxel	CR	NO
24	P#31	-	-	Docetaxel	PR	NO
25	P#32	-	+	Docetaxel	PR	YES
26	P#33	-	-	Abiraterone	PR	YES
27	P#34	-	-	Abiraterone	PD	YES
28	P#35	-	-	Docetaxel	PR	YES
29	P#36	-	-	Docetaxel	PR	YES
30	P#37	+	-	Enzalutamide	PD	YES
31	P#38	+	+	Docetaxel	PR	YES
32	P#39	+	+	Docetaxel	PD	YES
33	P#40	-	-	Enzalutamide	CR	NO
34	P#41	+	+	Docetaxel	PR	YES
35	P#42	-	+	Docetaxel	PR	YES
36	P#43	-	-	Docetaxel	PR	NO
37	P#44	-	+	Abiraterone	PD	YES
38	P#46	-	-	Abiraterone	CR	NO
39	P#47	+	-	Docetaxel	PR	YES
40	P#52	-	-	Abiraterone	PD	NO
41	P#53	+	+	Docetaxel	CR	NO
42	P#54	-	-	Abiraterone	CR	YES

43	P#56	+	-	Enzalutamide	PR	YES
44	P#57	+	-	Abiraterone	PR	NO

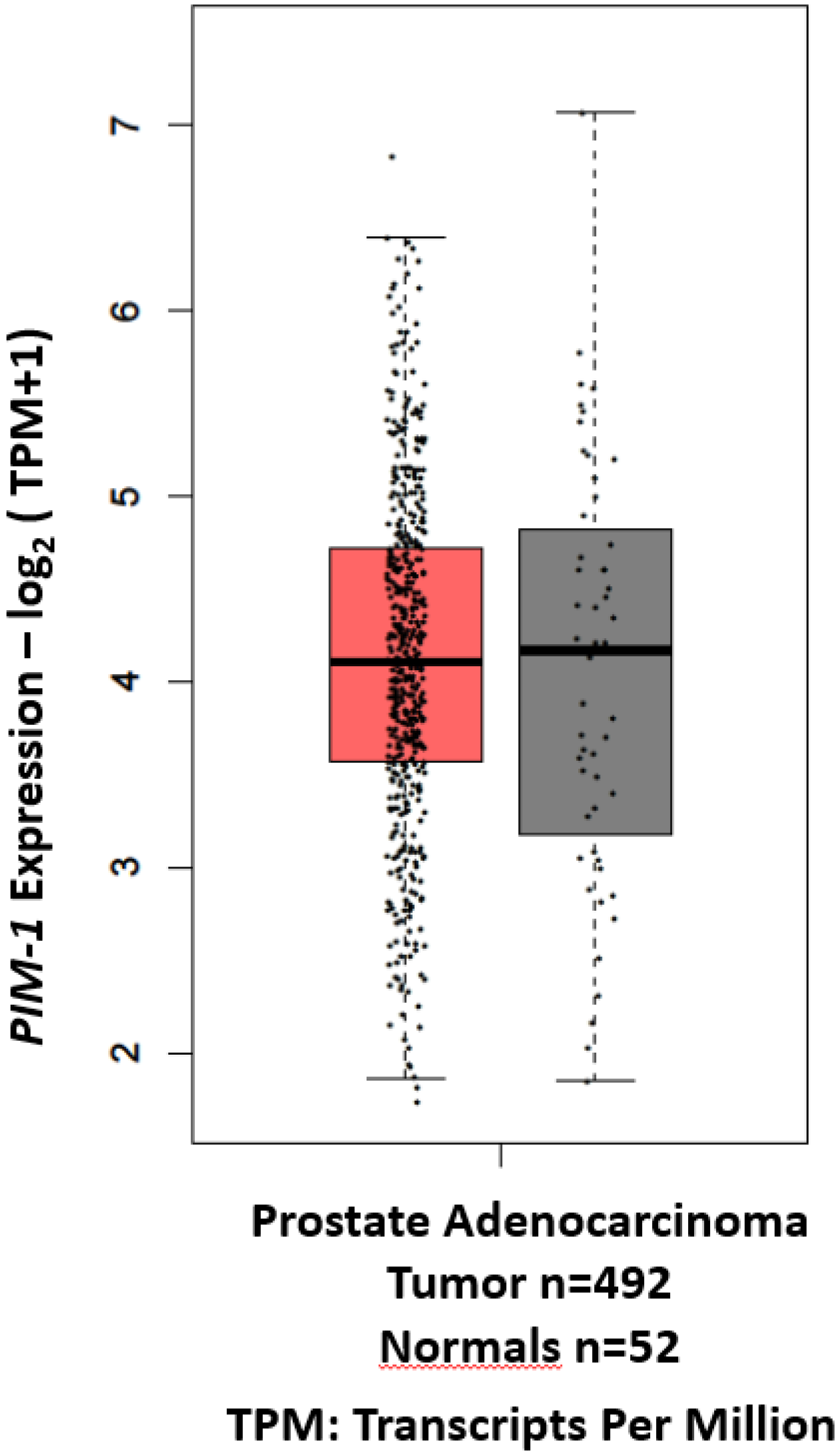

Figure S1. *PIM-1* expression in Prostate Adenocarcinoma Tumors (n = 492) and normal prostate tissues (n = 52) according to the TCGA.
